# MicroRNAs and long non-coding RNAs: prospects in diagnostics and therapy of cancer

**DOI:** 10.2478/raon-2013-0062

**Published:** 2013-10-08

**Authors:** Nina Hauptman, Damjan Glavac

**Affiliations:** Department of Molecular Genetics, Institute of Pathology, Medical Faculty, University of Ljubljana, Slovenia

**Keywords:** microRNAs, long non-coding RNAs, diagnosis, therapy, biomarker

## Abstract

**Background:**

Non-coding RNAs (ncRNAs) are key regulatory molecules in cellular processes, and are potential biomarkers in many diseases. Currently, microRNAs and long non-coding RNAs are being pursued as diagnostic and prognostic biomarkers, and as therapeutic tools in cancer, since their expression profiling is able to distinguish different cancer types and classify their sub-types.

**Conclusions:**

There are numerous studies confirming involvement of ncRNAs in cancer initiation, development and progression, but have only been recently identified as new diagnostic and prognostic tools. This can be beneficial in future medical cancer treatment options, since ncRNAs are natural antisense interactors included in regulation of many genes connected to survival and proliferation. Research is directed in development of useful markers for diagnosis and prognosis in cancer and in developing new RNA-based cancer therapies, of which some are already in clinical trials.

## Introduction

Cancer is one of the leading causes of death in the world, following deaths by cardiovascular and infectious disease. Although cancer is widely researched there is still lack of early detection techniques. For detecting early stage tumors and their precise characterization before and after treatment, biomarkers could be used, which consequently could lower the mortality rate.^1^ Research for suitable biomarkers for diagnosis and prognosis is wide-spread, and lately directed into detection in body fluids. For this purpose extensive research in the field of non-coding RNAs (ncRNAs) is conducted.

**FIGURE 1.**
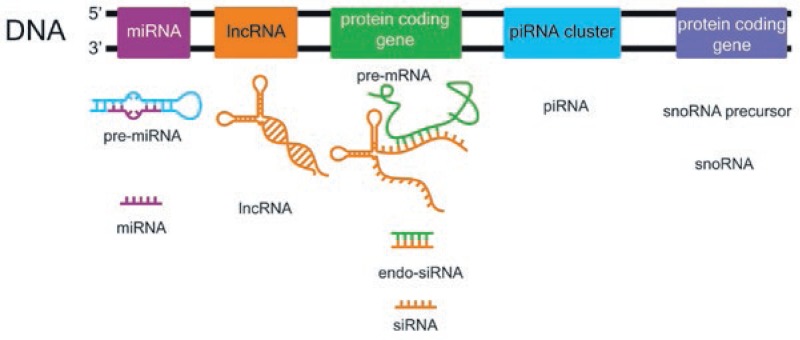
Schematic presentation of ncRNAs biogenesis.

RNA used to be considered the messenger between the gene and the protein encoded by this gene.^2^,^3^ The minority of the transcripts are protein coding (1.5%), and the rest used to be referred as “dark matter”, now known to be the ncRNA transcripts. Recent transcriptional analyses of genome estimate that ncRNA sequences are the most transcribed ones.^4^,^5^ The group of ncRNAs is quite diverse and complex. It is divided into ribosomal RNAs (rRNAs), transfer RNAs (tRNAs), micro-RNAs (miRNAs), long non-coding RNAs (lncRNAs), small nucleolar RNAs (snoRNAs), small interfering RNAs (siRNAs), small nuclear RNAs (snRNAs), and piwi-interacting RNAs (piRNAs) (Figure 1).^6^

MicroRNAs (miRNAs) are ∼22 nt long RNA molecules and are involved in post-transcriptional regulation. MiRNAs regulate over 30% of messenger RNAs (mRNAs), mainly through the negative regulation of gene expression, where miRNA bind to regions of mRNA, blocking the translation or completely degrading mRNAs.^7^ It is established that miRNA are included in cellular differentiation, development, proliferation and apoptosis, where they play an important role. In cancer these processes are deregulated, meaning that miRNA are involved in carcinogenesis, and could contribute to the initiation and progression of cancer.^8^ Tumor specific miRNA have a potential of becoming cancer biomarkers, since their expression profile can be more specific for determining the classification, diagnosis, and progression in cancer.^9^

LncRNA are classified as over 200 nt long transcripts that lack functionally open reading frame. They are involved in cellular differentiation and proliferation. The mechanisms through which they act are molecular scaffolds, which are involved in transcriptional machinery, as post-transcriptional regulators of splicing or as molecular decoys for miRNA.^4^,^10^ The lncRNA research is a new field emerging in molecular genetics, therefore only a small number of lncRNA were characterized. Comparing to miRNA, lncRNA studies are scarcer, nonetheless some promising evidence of using lncRNA as biomarkers for diagnosis and prognosis exist.

PiRNA are a class of regulatory small non-coding RNAs, 23–29 nt in length, which form the piRNA-induced silencing complex in the germ line of many animal species. PiRNA are specifically associated with PIWI proteins, which are germline-specific members of AGO protein family. The main function of piRNAs is defence against transposable elements in germ cells, and this role is highly conserved across animal species. Transposable elements threaten the genomic integrity of the host. PiRNAs and their interacting proteins have important role in cellular processes, and some of them are potential regulators of cancer cell development.^11^

SnoRNAs are 60–300 nucleotides in length and are predominantly found in nucleus. Their classical function is connected to post-transcriptional modification of ribosomal RNAs and some spliceosomal RNAs. These modifications are necessary for efficient and accurate production of ribosomes.^12^ Modification of ribosome biogenesis has been implicated in cancer development, which indicates snoRNAs might contribute to cancer, although this area needs further research.^12^,^13^

SiRNA are usually 19–23 nucleotides in length, which are known to guide silencing of target mRNA by directing the RNA-induced silencing complex to mediate site-specific cleavage, and destruction of targeted mRNA.^14^ Genes associated with cancer are a potential target of siRNAs, their potential is in inhibition and therapeutics. 14–17

In this review we will highlight the potential of miRNA and lncRNA for diagnosis and therapy, focusing on specific and sensitive biomarkers and their availability in body fluids. Additionally we will address the therapeutic benefits of miRNA and strategies of delivery to damaged tissues.

## Potential in diagnostics

Biomarkers are biological indicators of disease states, used to classify cancer types or subtypes.^18^ Effective and clinically relevant biomarkers are important for subsequent patient’s treatment.^19^ The research on detection of both miRNAs and lncRNAs is orientated toward their detection in body fluids. Comparing to mRNA, the level of expressions of either miRNA or lncRNA may be a better tool for indication of a certain disease. Furthermore, this can be diagnostically applicable when a distinctly specific pattern of expression for a certain disease exists.

One of the reasons of extensive research done on miRNAs connected to cancer is the possibility of conducting research on formalin fixed paraffin embedded (FFPE) samples. Due to their small length, miRNAs are not affected by formalin fixation and degradation over time like longer RNA molecules, such as mRNA and lncRNA, where fresh frozen samples are needed.^20^–^22^

### MiRNA diagnostic

The most commonly observed miRNA, which is up-regulated in human cancers, is miR-21 (Table 1). Overexpression was observed in breast, lung, prostate and other cancers, where it was shown to increase cell proliferation and invasion, and its suppression led to decrease in the cell proliferation, invasion, and induced apoptosis.^23^–^25^

Another miRNA up-regulated in breast, lung, pancreatic and other cancers is miR-155, which overexpression is also associated with tumorigenesis in lymphoma.^52^ Also in blood samples these two miRNAs are the most deregulated. Other miRNAs do not overlap in the cancer type groups either in tissue or blood samples. The overlap between the tissue and blood samples of the same cancer type was observed in prostate cancer, where miRNA-141 is expressed in tissue and patients sera, and could differentiate between patients with cancer and healthy controls.^51^ Another example is observed in plasma of patients with colorectal cancer (CRC), where levels of miR-29a and miR-92a are able to distinguish advanced adenomas and negative controls.^53^ In the research of circulating miR-141 in 102 plasma samples, a significant correlation to colon cancer stage IV was determined.^54^ The accuracy was further improved by combining the levels of miR-141 to carcinoembryonic antigen marker. For more accurate diagnostics, expression levels of several miRNAs should be monitored. Expression of 47 miRNAs in 101 FFPE samples of primary cancers and metastasis was evaluated, determining the tissue of origin. The identification of tissue was 100% for primary cancers and 78% for metastases. The accuracy remained high for independent sample validation.^55^ miRNA expression arrays can be utilized, when the other established clinical tests are inconclusive.

### LncRNA diagnostic

lncRNA is a fast growing field of research and many discovered lncRNA are deregulated in cancer (Table 2).

HOTAIR interacts with polycomb repressor complex PRC2, which causes the transcriptional silencing of several metastasis suppressor genes located in *HOXD* locus on chromosome 2.^56^ Elevated expression of HOTAIR was observed in primary and metastatic breast cancer compared to normal tissue. The high expression of HOTAIR is also correlated to metastasis and poor survival rate.^56^ HOTAIR can be a potential biomarker for the existence of lymph node metastasis in hepatocelular carcinoma (HCC).^57^

ANRIL activates two polycomb repressor complexes, PRC1 and PRC2, which results in chromatin reorganization, silencing the INK4b-ARFINK4a locus encoding tumor suppressor genes, involved in cell cycle inhibition, and stress-induced apoptosis. Overexpression of ANRIL in prostate cancer has shown silencing of INK4b-ARF-INK4a and p15/CDKN2B by heterochromatin reformation.^58^,^87^

MALAT1 is widely expressed in normal human tissues and is found to be up-regulated in a variety of human cancers of the breast, prostate, colon, liver and uterus.^75^,^76^ The MALAT1 locus is located at 11q13.1 and was found to harbour chromosomal translocation break points associated with cancer.^88^ It has been shown that increased expression of MALAT1 can be used as a prognostic marker for HCC patients following liver transplantation.^89^

*H19* and the insulin-like growing factor 2 (IGF2) are imprinted, and expressed from the maternal allele, and from parental allele, respectively.^62^,^68^ The loss of imprinting results in misexpression of H19 and was observed in many tumors including hepatocellular and bladder cancer.^64^*c-MYC* induces the expression of H19 in different cell types where H19 potentiates tumorigenesis.^68^

LncRNA MEG3 is a transcript of the maternally imprinted gene. In normal pituitary cells MEG3 is expressed, the loss of expression is observed in pituitary adenomas and the majority of meningiomas and meningioma cell lines. MEG3 activates regulation of tumor suppressor protein p53.^77^,^78^

Growth Arrest-Specific 5 (GAS5) functions as a starvation or growth arrest-linked riborepressor for the glucocorticoid receptors by binding to their DNA binding domain inhibiting the association of these receptors with their DNA recognition sequence. This suppresses the induction of several responsive genes including the gene encoding cellular inhibitor of apoptosis 2 (cIAP2), reducing cell metabolism and synthesizes cells to apoptosis.^90^ GAS5 can induce apoptosis directly or indirectly in the prostate and breast cancer cell lines, where it was shown that GAS5 has a significantly lower expression in breast cancers compared to normal breast epithelial tissues.^86^

One of the lncRNA utilized in a clinical test is prostate cancer associated (PCA3), which is a prostate cancer specific lncRNA. It can be detected in urine samples obtained after a prostatic massage.^91^,^92^ Studies, comparing the levels of PCA3 to current biomarker prostate specific antigen (PSA), were conducted, showing that PCA3 has higher specificity than PSA, reducing the number of biopsies. Also PCA3 levels correlate better to identification of disease, since PSA levels can be also elevated due to inflammatory reasons. The accuracy was improved when profiling of both PCA3 and PSA in blood was performed.^93^

There are two lncRNA connected to HCC, highly up-regulated in liver cancer (HULC) and HOTAIR. HULC is detected in peripheral blood cells and therefore has a potential as a biomarker.^72^ HOTAIR has also been correlated to HCC and has potential to become a biomarker for lymph node metastasis and tumor recurrence in HCC patients’ undergone a liver transplant.^57^,^70^

Clinical trials on biomarkers are mostly performed on specimens that are easily obtainable, such as blood or urine, and present little discomfort to patients, where on the other hand trials are rare on tumor tissue, due to the specimen unavailability. The detection of early stage disease in body fluids is ideal for patients, due to its non-invasive nature. Still many questions persist, like stability of the circulating molecules, and their stability in the progression of disease. There is also evidence of some specific expression in cancers, but with the on-going research on this topic there will be more evidence of involvement of lncRNA in cancer.^71^,^93^

## Potential of therapy

After proving many miRNA and lncRNA are deregulated in cancer, the research now focuses on their role as therapeutic targets.^94^

**FIGURE 2.**
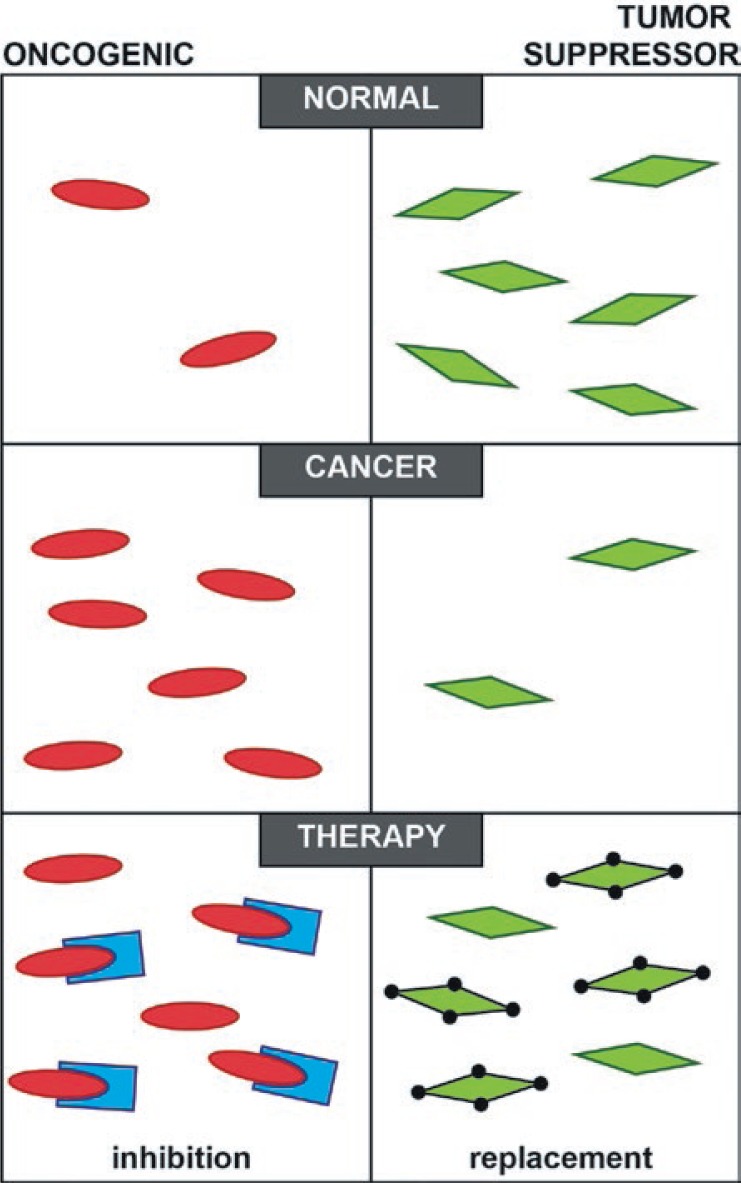
Schematic presentation of oncogenic and tumor suppressor miRNAs in normal and cancer cell and the potential of modifying state in cancer cell with therapy.

MiRNAs involved and deregulated in cancer are divided into tumor suppressor and oncogenic miRNAs. Oncogenic miRNAs are overexpressed in cancer, downregulating tumor suppressor genes.^95^ To reverse the oncogenic miRNA expression they have to be inhibited to relieve their targets. This can be achieved by introducing mRNAs targeting specific miRNAs or by using antisense single-stranded oligonucleotides complementary to miRNA, acting as miRNA sponges and miRNA antagonists, respectively.^96^–^98^ On the other hand tumor suppressor miRNAs are under expressed in the cancer, their role being down-regulation of oncogenes.^95^,^99^ To restore the levels of tumor suppressor miRNAs the replacement therapy of mimics miRNA or DNA coding for specific miRNAs is needed.^96^,^100^ This is schematically presented in Figure 2.

Inhibition of oncogenic miRNAs has been widely researched through siRNA-based therapeutic modalities, and antisense oligonucleotides, which have been a straightforward approach relieving repressed targets of miRNA.^101^,^102^ Antisense oligo-nucleotides can be designed to potentially block several steps during the biogenesis and action of miRNA, miRNA processing or miRNA pairing with targeted mRNA. *In vitro* and *in vivo* mice studies used modified antisense oligonucleotides to inhibit tumor proliferation, migration, invasion, and apoptosis.^96^ Antisense oligonucleotide targeting miR-21 in *in vitro* and *in vivo* xenograft model resulted in the inhibition of breast cancer cell growth, inhibited cell proliferation, and increased apoptosis.^103^ Besides antisense oligonucleotide inhibition, miRNA sponges as another technique to effectively lower the levels of miRNA has been used, where targeted sequence is cloned in multiple copies, and upon transfection into a tumor cell should act as a sponge for the miRNA and relieve its natural target.^104^ In breast cancer cell lines, a miRNA sponge trapping up-regulated miR-9 connected to cancer metastasis effectively reduced invasiveness of the tumor cells.^105^

The replacement therapy for down-regulated tumor suppressor miRNA is administration of synthetic miRNA. Tumor suppressor let-7 miRNA, known to be associated with many tumors, was delivered intratumorally in a mouse model of non-small-cell lung cancer, which led to reduction of tumor burden.^106^ Several studies suggest that let-7 acts through direct repression of *KRAS* and *c-MYC* oncogenes.^107^ Another deregulated miRNA associated with several cancers is miR-34. Through transfection or lentiviral-mediated delivery of mimic miR-34 to cancer cells, the cell-cycle arrest, apoptosis and reduction in tumor size was observed.^108^

It is observed, in both EU and US, a large increase of patents connected to miRNA. Many miRNA based therapeutics is either in preclinical or clinical trial phase. In cancer treatment Mirna Therapeutics has developed miRNA mimic therapeutics for miR-34 (phase I) and let-7 (preclinical).

While many targeting strategies are implied to reverse the levels of miRNA, for lncRNA these strategies are still being developed. In principle the same strategies as for miRNA could be used, like introducing molecules designed to target lncRNA to lower the expression levels or disrupt the lncRNA in structural or functional way.

There is evidence that the expression can be lowered through RNAi technology, degradation by RnaseH or by genomic integration of RNA destabilizing elements.^109^ Modifying the expression levels can represent some difficulty due to possible secondary structure of lncRNA. Inactivation of lncRNA is also possible through inhibition of active site via small molecule inhibitors. To be able to do this the molecular function needs to be known, which for most lncRNA is still elusive. It is also possible to disrupt the structure of lncRNA. Due to their length it is presumed some secondary structures exist. With the use of specially designed small molecules this structures would be disrupted leading to lncRNA loss of function. The potential of using specific therapeutics that would enable the mimicking or inhibition of certain non-coding RNA is promising and enormous.^110^

To reverse the levels of disrupted lncRNA in cancer a replacement therapy is also an option. Some strategies of delivery are being explored. The use of lncRNA H19 specific expression in tumors has been explored through a plasmid delivery. Intratumoral delivery of plasmid, which carries the gene for the A subunit of diphtheria toxin under the regulation of H19 promoter, induces high expression of diphtheria toxin, which results in reduced tumor size.^111^

## Conclusions

Studies of miRNA and lncRNA have highlighted the importance of non-coding part of human genome. Of all lncRNA only few have been well characterized. Research also shows they have important function in cancer initiation, progression and metastasis. Further expression patterns in cancer will improve diagnosis and prognosis of cancer. With more functional and structural studies the potential of lncRNA therapies will be seen.

MiRNA as regulators of multiple genes promise a great potential in therapeutics and a switch from one drug one target to one drug multiple target therapy. Although there were great advances made in replacement and inhibitory strategies there are still challenges that include stability, safety and delivery of the chosen therapeutics. For therapeutics to become a successful application, the drug needs to be delivered in a way that ensures the stability of the molecules’ transport to the appropriate cells.

## Figures and Tables

**TABLE 1.** Potential diagnostic miRNA biomarkers in tissue and blood samples

**Cancer**	**Tissue samples (FFPE) (expression status ↑, ↓)**	**Reference**	**Blood samples (expression status ↑, ↓)**	**Reference**
Breast cancer	miR-21↑, miR-155 ↑, miR-191 ↑, miR-196a ↑, miR-125b ↓, miR-221 ↓, let-7a ↓, miR-145 ↓, miR-205 ↓	23, 26	Serum: miR-10b ↑, miR-34a ↑, miR-155 ↑, miR-21 ↑, miR-106a ↑, miR-155 ↑, miR-126 ↓, miR-199a ↓, miR-335 ↓ Whole blood: miR-195 ↑	27–29
Lung cancer	miR-21, miR-205	30	Serum: miR-10b ↑, miR-155 ↑	31
Gastric cancer	miR-106a ↑, miR-31 ↓	32, 33	Serum: miR-10a ↑, miR-22 ↑, miR-100 ↑, miR-148b ↑, miR-223 ↑, miR-133a ↑, miR-127-3p ↑, miR-1 ↑, miR-20a ↑, miR-27a ↑, miR-34 ↑, miR-423-5p ↑	34, 35
Pancreatic cancer	miR-452 ↑, miR-105 ↑, miR-127 ↑, miR-518a-2 ↑, miR-187 ↑, miR-30a-3p ↑, miR-21 ↑, miR-155 ↑, miR-221 ↑, miR-222 ↑, let-7a ↑	36–38	Serum: miR-21 ↑, miR-155 ↑, miR-196a ↑ Plasma: miR-21 ↑, miR-155 ↑, miR-196a ↑, miR-210 ↑	39–41
Prostate cancer	miR-125b ↑, miR-15a ↓, miR-16 ↓, miR-184 ↑, miR-146a ↓, miR-203 ↓, miR-34c ↓, miR-141 ↑	42–46	Serum: miR-141 ↑, miR-21 ↑, miR-141 ↑, miR-221 ↑, miR-375 ↑	47–51

**TABLE 2.** lncRNA deregulated in cancer

**Name**	**Size (kb)**	**Cancer Type**	**Expression**	**Reference**
**ANRIL**	∼3.9	Prostate, leukemia	↑	58
**BC200**	0.2	Breast, cervix, esophagus, lung, ovary, parotid, tongue	↑	59, 60
**PRNCR1**	13	Prostate		61
**H19**	2.3	Bladder, lung, liver, breast, esophagus, choriocarcinoma, colorectal cancer		62–68
**HOTAIR**	2.2	Breast, hepatocellular	↑	56, 57, 69, 70
**HULC**	∼0.5	Hepatocellular	↑	71, 72
**MALAT1**	7.5	Breast, prostate, colon, liver, uterus	↑	73–76
**MEG3**	1.6	Brain	↓	77, 78
**PTNEP1**	3.9	Prostate		79
**Spry4-it1**	∼0.7	Melanoma	↑	80
**SRA**	1.965	Breast, uterus, ovary	↓	81, 82
**UCA1/CUDR**	1.4, 2.2, 2.7	Bladder, colon, cervix, lung, thyroid, liver, breast, esophagus, stomach	↑	83, 84
**PCA3**	0.6–4	Prostate	↑	85
**GAS5**	isoforms	Breast	↓	86
